# The status of p53 affects the efficacy of PLK1 inhibitor BI6727 in prostate cancer cells

**DOI:** 10.3389/fcell.2025.1602693

**Published:** 2025-07-30

**Authors:** Wenjing Li, Ying Wang, Wenzheng Guo, Donghua Wen

**Affiliations:** Department of Laboratory Medicine, Shanghai East Hospital, Tongji University School of Medicine, Shanghai, China

**Keywords:** prostate cancer, PLK1, PLK1 inhibitor, BI6727, p53

## Abstract

As there are no effective treatments for advanced prostate cancer, exploring new therapies is crucial. BI6727(Volasertib), a PLK1 inhibitor, shows great promise as an anti-cancer drug. However, despite advancing to phase II and III trials in other cancers, BI6727 has shown limited anti-tumor activity in prostate cancer, making it crucial to investigate the underlying reasons for this discrepancy. In this study, we found that the status of p53 affects the sensitivity of prostate cancer cells to BI6727. Prostate cancer cells PC3 (long-term loss of p53 expression), DU145 (expressing mutant-type p53) and LNCaP (expressing wild-type p53) were treated with BI6727, respectively. It was found that PC3 cells were more sensitive to BI6727 when wild-type p53 was introduced into these cancer cells; while apoptosis induced by BI6727 was reduced after knockdown of p53 in LNCaP cells. In additional, in DU145 cells, the presence of points mutation in p53 exerted a dominant negative effect, attenuating BI6727-induced apoptosis. Further analysis revealed that missense mutations in the *P53* gene are widespread in prostate cancer patients. Mechanistically, BI6727 reduces the degradation of Topors, thereby increasing the stability of p53 by reducing its ubiquitination. This ultimately influences the sensitivity of prostate cancer cells with different p53 statuses to BI6727.In summary, this study identifies p53 as a key factor limiting the clinical efficacy of BI6727 in prostate cancer cells.

## 1 Introduction

Prostate cancer is the second most common cancer worldwide and the fifth leading cause of cancer-related death in men in 2022. It accounts for 14.2% of all male cancer cases and 7.3% of male cancer-related deaths globally (Bray et al., 2024). In advanced prostate cancer, resistance and disease progression are common, even with high-toxicity chemotherapy (Tilki et al., 2024). Therefore, the identification of new therapeutic agents for prostate cancer is critical. PLK1 is overexpressed in various cancers, including prostate cancer, ovarian cancer, small cell lung cancer, and pancreatic cancer (Wang et al., 2022; Zhang et al., 2022a; Zhang et al., 2024; Iliaki et al., 2021), and its expression level is often associated with poor prognosis (Iliaki et al., 2021). High expression of PLK1 drives tumorigenesis, and its overexpression is directly associated with the onset of prostate cancer (Gheghiani et al., 2021; Wu et al., 2016). Currently, among over ten PLK1 specific inhibitors, at least four (BI2536, BI6727, GSK461364, and NMS-1286937) have been evaluated in clinical trials (Su et al., 2022). As a typical kinase domain inhibitor, BI6727 received FDA’s “Breakthrough Therapy Designation” in 2013 (Zhang J. et al., 2022). BI6727 has shown promising clinical activity in advanced solid tumors such as ovarian cancer, non-small cell lung cancer, urothelial carcinoma, and leukemia, and has progressed to phase II and even phase III trials (Döhner et al., 2021; Pujade-Lauraine et al., 2016; Stadler et al., 2014). However, in prostate cancer, despite strong preclinical anti-tumor activity, BI6727 has shown only minimal anti-tumor efficacy in phase I trials (Schöffski et al., 2012). Therefore, it is crucial to investigate the factors influencing its clinical efficacy in prostate cancer.

The PLK1-p53 axis is a complex signaling pathway, with reciprocal interactions between the two (Meitinger et al., 2024; Li et al., 2025). PLK1 depletion can activate the p53 signaling pathway (Zhang et al., 2022c; Jia et al., 2019), while p53 regulates the expression of PLK1 through various mechanisms (McKenzie et al., 2010; Wang et al., 2020). Studies have shown that p53 knockdown or damage in colorectal cancer, large cell lung cancer, and breast epithelial cells increases sensitivity to PLK1 inhibitor-induced cytotoxicity (Guan et al., 2005; Degenhardt et al., 2010; Sur et al., 2009). Therefore, researchers suggest that the inactivation of functional (wild-type) p53 makes cells more responsive to PLK1 inhibitors. Some studies suggest that in cell lines such as colorectal cancer HCT116, cervical cancer HeLa, lung cancer A549, breast cancer MCF7, and osteosarcoma U2OS(24-26), wild-type p53 renders tumor cells more sensitive to PLK1 inhibitors and enhances the apoptotic effects induced by PLK1 inhibition. However, other studies have shown that the p53 status does not alter tumor cell sensitivity to PLK1 inhibitors (Moison et al., 2019), and that PLK1 inhibitors selectively target cells with mutant p53 rather than wild-type p53 cells (Li Y. et al., 2020). Previous studies have reported inconsistent effects of p53 on the efficacy of BI6727. In the present study, we systematically investigated the effect of different p53 states on apoptosis induced by the PLK1 inhibitor BI6727 in prostate cancer cells. We used PC3 (long-term loss of p53 expression), LNCaP(expressing wild-type p53), and DU145 (expressing mutant-type p53) (Doulabi et al., 2024) as natural research models to study the effect of p53 status on BI6727. We also investigated the mutational status of p53 in prostate cancer patients in this study.

## 2 Materials and methods

### 2.1 Cell culture and generation of cell lines

The PC3, LNCaP, and DU145 cell lines were sourced from the Department of Laboratory Medicine, Shanghai East Hospital. DU145 and PC3 cells were cultured in DMEM (BasaIMedia) and RPMI 1640 (BasaIMedia), respectively, each supplemented with 10% fetal bovine serum (FBS). The LNCaP cells were cultured in RPMI 1640 medium supplemented with 15% fetal bovine serum (FBS). All cells were grown in normal culture medium for at least 1 day prior to each experiment.

### 2.2 Lentivirus preparation and infection

To prepare the lentivirus, plasmids containing p53 (MIAOLING BIOLOGY) and packaging (psPAX2) and envelope (pMD2.G) vectors were transfected into 293T cells using Hieff TransTM Liposomal Transfection Reagent (Yeasen) according to the manufacturer’s instructions. Approximately 16 h later, the transfection medium was replaced by the fresh complete medium. After 48 h of transfection, the supernatant was collected, filtered through a 0.45-μm filter, and ultracentrifuged at 28,000 rpm for 2 h at 4°C. The recovered viral particles were used to infect cells in the presence of 8 μg/mL polybrene.

### 2.3 Western blot and antibodies

A total of 1 × 10^7^ cells were harvested and washed with PBS and lysed with lysis buffer (50 mM Tris-HCl, pH 6.8, 100 mM DTT, 2% SDS, 10% glycerol). The cell lysates were centrifuged at 16,000 g for 10 min, and protein concentration was determined. Equal amounts of protein were loaded onto 8%–15% SDS-PAGE gels, followed by electrophoresis and transfer to a nitrocellulose membrane. After blocking the membrane with 5% non-fat milk in PBS for 1 h, the membrane was incubated with primary antibodies against PLK1 (Millipore, 05-844), PARP (Cell Signaling Technology, 9532S), Caspase-3 (Cell Signaling Technology, 9661S), p53 (Beyotime Biotechnology, AF0255 and AF7671), ubiquitin (Proteintech, 10201-2-AP), Topors (Santa Cruze,sc-101182),α-tubulin (Sigma-Aldrich, ABT171), or β-actin (Santa Cruze,sc-47778). Signals were detected using a chemiluminescence HRP detection kit (Zetalife, 310208) according to the manufacturer’s instructions.

### 2.4 Colony formation assay

Seed 2,000 cells/well into a 6-well plate and incubated for 14 days. Fix in 4% paraformaldehyde for 30 min, stain with crystal violet for 30 min, wash, dry, and capture images.

### 2.5 Cell counting kit-8 assay

Following the manufacturer’s instructions, 20 µL of CCK-8 solution (Beyotime Biotechnology) was added to each well of a 96-well plate containing 2 × 10^3^ cells. After incubation for 2 h at 37°C, the absorbance was measured at 450 nm.

### 2.6 Transient transfection

p53 overexpression and knockdown were achieved by transient transfection of cells with pcDNA-P53 (MIAOLING BIOLOGY) and P53 siRNA (RIBOBIO), respectively. Cells were transfected with negative control or pcDNA-P53 or siP53 using Hieff TransTM Liposomal Transfection Reagent (Yeasen) according to the manufacturer’s protocol. siNC refers to a negative control siRNA with no homologous sequence in the human or mouse transcriptome. The target sequences for the three P53 siRNAs used in this study are as follows:

siP53-1: 5′-GTACCACCATCCACTACAA -3’.

siP53-2: 5′-AGAGAATCTCCGCAAGAAA -3’.

siP53-3: 5′-GGAGTATTTGGATGACAGA -3’.

### 2.7 Data acquisition and processing

Data on STAR counts, mutation MAF, and corresponding clinical information for prostate cancer were obtained from the TCGA database (https://portal.gdc.cancer.gov). Subsequently, data were extracted from the STAR counts in TPM format, followed by log2 (TPM+1) normalization. After filtering for samples that contained RNA sequencing data, MAF mutation data, and clinical information, a total of 494 samples were included for further analysis. Somatic mutation data from prostate cancer patients were obtained and visualized utilizing the maftools package within the R software environment. Statistical analyses were conducted using R software version 4.0.3. Results were considered statistically significant at a *P*-value threshold of less than 0.05.

### 2.8 Timer2.0 analysis

The Gene_Mutation module, utilizing Timer2.0 (http://timer.comp-genomics.org/) (Li T. et al., 2020), was used to compare the expression levels of the *P53* gene in both wild-type and mutant states of the PLK1 gene.

### 2.9 Statistical analyses

Statistical analysis was performed using Excel or GraphPad Prism 9.5 software. Quantitative data are presented as mean ± SEM, and *p* < 0.05 was considered statistically significant.

### 2.10 Immunoprecipitate

Cells were harvested and washed with pre-chilled PBS, then lysed in NETN buffer (150 mM NaCl, 1 mM EDTA, 20 mM Tris, 0.5% Nonidet P-40) supplemented with protease inhibitors (Beyotime Biotechnology). The lysates were sonicated and clarified by centrifugation at 12,000 × g for 5 min at 4°C, repeated three times.After centrifugation, the supernatant was incubated with the appropriate antibody at 4°C for 16 h, followed by incubation with Protein A/G PLUS-Agarose beads (Santa Cruze,sc-2003) for 2 h at 4°C. The beads were then collected by centrifugation at 2,000 × g for 5 min, and the supernatant was discarded. Beads were washed at least five times with NETN lysis buffer and then lysed with lysis buffer.

## 3 Results

### 3.1 Introduction of wild-type p53 into PC3 cells enhances the BI6727-induced apoptosis

**FIGURE 1:**
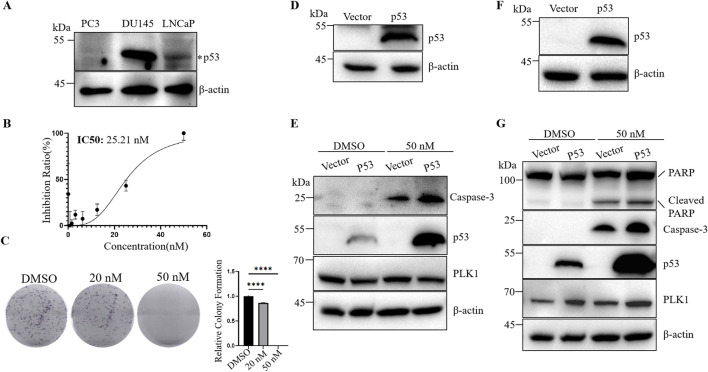
Wild-type p53 promotes BI6727-induced apoptosis in PC3 cells. **(A)** Cell lysates from PC3, DU145, and LNCaP cells were collected and analyzed by Western blot to detect endogenous p53 protein levels. **(B)** CCK-8 assay to evaluate the effect of BI6727 on PC3 cell growth. **(C)** PC3 cells were treated with DMSO, 20 nM, or 50 nM BI6727, and colony formation was assessed. **(D)** p53 expression was detected in PC3 cells by Western blot after transient transfection. **(E)** After treating PC3-Vector and PC3-p53 cells with DMSO and 50 nM BI6727 for 48 h, transient transfection cells were collected to detect activation form of caspase-3 by Western blot. **(F)** A stable p53-expressing PC3 cell line was constructed, and p53 protein expression was detected by Western blot. **(G)** After treating the stable PC3-Vector and PC3-p53 cells with DMSO or 50 nM BI6727 for 48 h, proteins were collected and detected by Western blot using specific antibodies.

Although many studies have investigated the effect of p53 on the efficacy of PLK1 inhibitors, the results have been inconsistent. Previous studies mainly used tumor cells with wild-type p53 and did not investigate the effect of p53 on BI6727-induced apoptosis in tumor cells with long-term p53 loss. PC3 cells are prostate cancer cells with long-term p53 loss due to *P53* gene mutation (Isaacs et al., 1991) (Figure 1A). BI6727 has been shown to exert cytotoxic effects on various tumor cells (Gjertsen and Schöffski, 2015). The analysis of CCK-8 assay showed that BI6727 could inhibit the proliferation of PC3 cells. The IC50 of BI6727 in PC3 cells was 25.21 nM (Figure 1B). Additionally, the colony formation assay showed decreased cell proliferation in PC3 cells treated with BI6727, especially in PC3 cells treated with 50 nM BI6727 (Figure 1C). In order to explore if p53 affects the sensitivity of PC3 to BI6727, p53 was successfully transiently expressed in PC3 cells (Figure 1D). After BI6727 treatment, the activation form of caspase-3 was higher in PC3-p53 cells than in PC3-Vector cells (Figure 1E). Then, the PC3 cell lines stably expressing Vector or wild-type p53 were established (Figure 1F). The same results were obtained. After BI6727 treatment, the activation form of caspase-3 was elevated and the cleavage of its substrate PARP increased (Figure 1G). These data indicate that, in PC3 cells with long-term p53 loss, the presence of functional p53 increased the sensitivity of them to the PLK1 inhibitors BI6727 (Zhu et al., 2002; Kruse and Gu, 2009; Ando et al., 2004).

### 3.2 Endogenous p53 knockdown weakens BI6727-induced apoptosis effect in LNCaP cells

**FIGURE 2:**
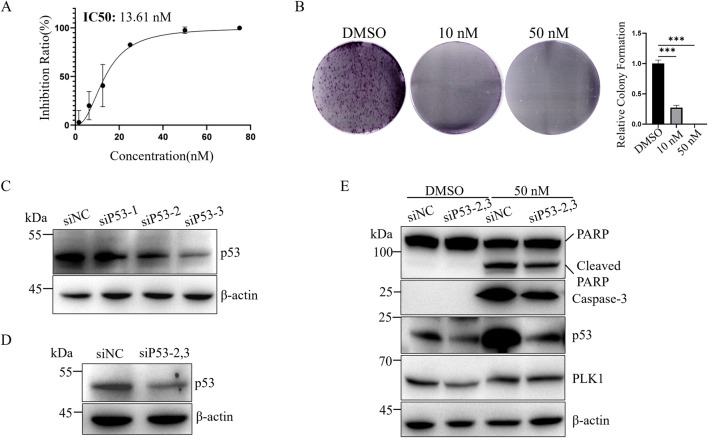
Knockdown of p53 weakens BI6727-induced apoptosis in LNCaP cells. **(A)** CCK-8 assay to assess the effect of BI6727 on LNCaP prostate cancer cell growth; **(B)** LNCaP cells were treated with DMSO, 20 nM, and 50 nM BI6727 to evaluate colony formation; **(C)** Cells were treated with 50 nM siNC, siP53-1,siP53-2 or siP53-3, and protein lysates were prepared to assess p53 protein levels; **(D)** LNCaP cells were treated with 50 nM control si-NC or siP53-2and siP53-3 (siP53-2,3) for 56 h **(E)** LNCaP cells were treated with siRNA siP53-2,3 for 16 h, then cultured in normal medium for 40 h. Afterward, PLK1 inhibitor BI6727, or DMSO, and cells were incubated for 48 h before collection and protein extraction for Western blot.

LNCaP cells are androgen-dependent prostate cancer cells with functional p53 (Isaacs et al., 1991; Hierowski et al., 1987) (Figure 1A). The IC50 of BI6727 in LNCaP cells is 13.61 nM (Figure 2A). As the concentration increased, BI6727 became more effective in inhibiting LNCaP cell proliferation, and at 50 nM, no colonies were formed (Figure 2B), consistent with PC3 cells. BI6727 exerts different levels of cytotoxicity in PC3 and LNCaP cells, with the IC50 in LNCaP cells being significantly lower than in PC3 cells. Since LNCaP cells express wild-type p53, we hypothesized that the presence of p53 may influence the cytotoxic effect of BI6727. Therefore, we knocked down endogenous p53 in LNCaP cells to investigate its role in BI6727-induced apoptosis. We used three different siP53s to knock down p53, with siP53-1 showing a much lower knockdown efficiency than siP53-2 and siP53-3 (Figure 2C). Therefore, in subsequent experiments, we used a 1:1 mixture of siP53-2 and siP53-3 (siP53-2,3) to knock down p53. The results showed that p53 expression was significantly reduced in LNCaP-siP53-2,3 cells compared to LNCaP-siNC (Figure 2D). Cells were treated with siP53-2,3 for 16 h and then cultured in normal medium for 40 h. The PLK1 inhibitor BI6727 or DMSO was then added, and cells were incubated for 48 h before harvesting and protein extraction for Western blot. After BI6727 treatment, p53 knockdown in LNCaP-siP53-2,3 cells significantly decreased the activation form of caspase-3 and downstream PARP cleavage (Figure 2E). Compared to the control, BI6727 treatment increased p53 expression in the cells (Figure 2E), which is consistent with reports that PLK1 inhibition induces apoptosis by increasing p53 expression and activating p53-dependent transcriptional activity (Wang et al., 2024). Therefore, BI6727 induces apoptosis through a p53-dependent pathway in wild-type p53 cells. Overall, in LNCaP cells, p53 promotes apoptosis induced by BI6727.

### 3.3 Knockdown of mutated p53 enhances BI6727-induced apoptosis in DU145 cells

**FIGURE 3:**
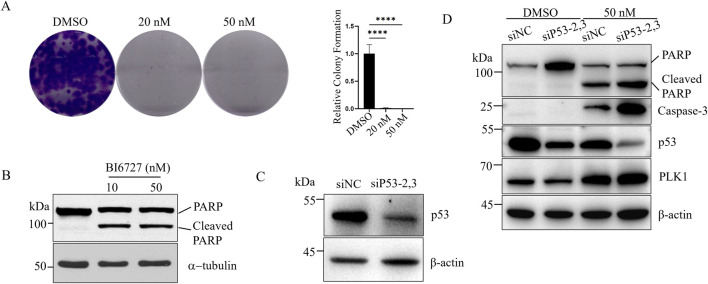
The expression of mutant p53 inhibits BI6727-induced apoptosis in DU145 cells. **(A)** DU145 cells were treated with DMSO, 20 nM, and 50 nM BI6727, and colony formation was assessed. **(B)** DU145 cells were treated with 10 nM and 50 nM BI6727 for 48 h, then collected and analyzed by Western blot using antiPARP antibody. **(C,D)** DU145 cells were treated with siP53-2,3 for 16 h, then cultured in normal medium for 40 h. Afterward, PLK1 inhibitor BI6727, or DMSO, and cells were incubated for 48 h before collection and protein extraction for Western blot using the indicated antibodies.

As we know, we can detect p53 in DU145 cells (Figure 1A), but it is a mutant. We next investigated the effect of BI6727 on p53-mutant DU145 prostate cancer cells. In the colony formation assay, with the concentration of BI6727 at 20 nM, only a few small clones were observed; and at 50 nM, there was nearly no colony formation (Figure 3A). To evaluate apoptosis effect of BI6727 on DU145 cells, cells were treated with 10 nM and 50 nM BI6727 for 48 h, PARP degradation was observed (Figure 3B). It has been suggested that mutant p53 is associated with the dominant negative effect of in DU145 cells (Zhu et al., 2002). DU145 cells were treated with siP53-2,3, the effect of siRNA was detected by Western blot (Figure 3C). The results showed that mutant p53 can be knocked down effectively in DU145 cells. When cells were treated with BI6727, the activation form of caspase-3 was increased and PARP cleavage was enhanced in DU145-siP53-2,3 cells compared to DU145-siNC (Figure 3D). In conclusion, in p53-mutant DU145 cells, the expression of mutant p53 reduces PLK1 inhibitor-induced apoptosis.

### 3.4 Mutated landscape of p53 in prostate cancer specimens

**FIGURE 4:**
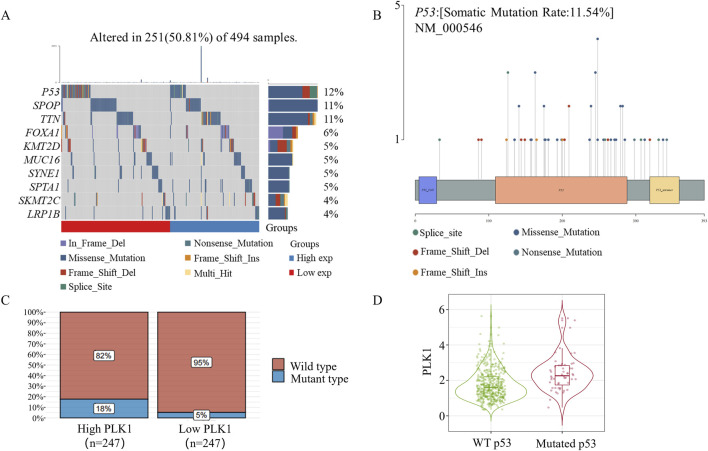
P53 Mutation in Prostate Cancer. **(A)** Somatic mutation landscape of 494 prostate cancer samples. The side bar chart shows the types and frequencies of gene mutations. The waterfall plot illustrates the mutation details of each gene across samples, with different colors at the bottom indicating specific mutation types. A small section above the legend shows the mutation burden count. **(B)** A lollipop plot showing the distribution of *P53* gene mutations, with the somatic mutation rates and names indicated in the figure’s title and subtitle. **(C)** P53 mRNA mutation distribution in 494 prostate cancer samples. Based on the median expression of PLK1, samples were grouped into high PLK1 expression and low PLK1 expression groups. **(D)** Timer2.0 analysis of *PLK1* gene expression differences under different p53 mutation statuses.

Considering that p53 mutation affects the effect of the PLK1 inhibitor BI6727 in inducing apoptosis in prostate cancer cells, we next investigated the mutation status of p53 in prostate cancer specimens. In this study, we performed a comprehensive gene mutation analysis on 494 prostate cancer samples and found that there is a 12% mutation frequency of the *P53* gene in prostate cancer (Figure 4A). Among the *P53* gene mutations, we observed various types, including missense mutations, splice site mutations, nonsense mutations, frameshift insertions, and frameshift deletions. The distribution of these mutations highlights the diversity and complexity of p53 in prostate cancer samples (Figure 4B). In addition, missense mutations were the most common type of p53 mutation. Further analysis of the cohort revealed that patients with high PLK1 expression also had a significantly increased mutation rate of p53 (Figure 4C). More intriguingly, in the prostate cancer specimens with p53 mutation, PLK1 expression was significantly increased (Figure 4D). Overall, the informatics assay showed that 12% of the prostate cancer specimens had a p53 mutation.

### 3.5 BI6727 selectively inhibits p53 ubiquitination, leading to increased p53 stability

**FIGURE 5:**
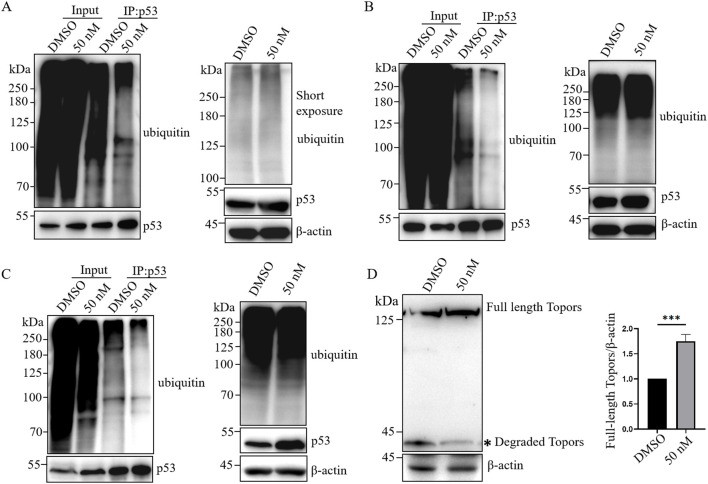
BI6727 reduces p53 ubiquitination. **(A)** 293T cells were co-transfected with p53 and HA-ubiquitin, treated with DMSO or 50 nM BI6727 for 48 h, followed by 10 μM MG132 for 4 h. Total ubiquitinated proteins were detected in lysates, and p53 was immunoprecipitated for detection of ubiquitinated p53 by Western blot. **(B,C)** 293T cells **(B)** and PC3 cells stably expressing p53 **(C)** were treated with DMSO or 50 nM BI6727 for 48 h, followed by 10 μM MG132 for 4 h. Total and p53-specific ubiquitination levels were examined as described in **(A)**. **(D)** 293T cells were treated with DMSO or 50 nM BI6727 for 48 h. Topors protein levels were analyzed by Western blot.* indicates degradation products of Topors.

Topors has been reported to ubiquitinate p53 and significantly reduce its protein levels (Rajendra et al., 2004). Phosphorylation of Topors by PLK1 enhances its activity, promoting p53 ubiquitination and subsequent degradation (Yang et al., 2009). Meanwhile, PLK1 phosphorylates Topors to promote its degradation (Yang et al., 2010).To determine whether the ubiquitin–proteasome system is involved in BI6727-induced cell death, we assessed both p53 ubiquitination and global protein ubiquitination. In 293T cells overexpressing exogenous p53 and ubiquitin, immunoblotting showed that BI6727 reduced p53 ubiquitination, with minimal effect on overall protein ubiquitination (Figure 5A).We also examined endogenous p53 ubiquitination in 293T cells and obtained similar results (Figure 5B). Furthermore, in PC3 prostate cancer cells stably expressing p53, BI6727 likewise reduced p53 ubiquitination with little effect on overall ubiquitination levels (Figure 5C).Since BI6727 reduced p53 ubiquitination, we next investigated whether this effect was mediated by Topors. We found that BI6727 treatment markedly attenuated Topors degradation, leading to increased Topors protein levels (Figure 5D). These findings suggest that the PLK1 inhibitor BI6727 enhances p53 stability by inhibiting Topors degradation and thereby reducing p53 ubiquitination.

## 4 Discussion

p53 is a critical tumor suppressor protein that regulates cell growth by promoting apoptosis (Kanapathipillai, 2018; Eliyahu et al., 1989; Finlay et al., 1989). Functional inactivation of p53 is the most common mutation observed in various cancers (Tornesello, 2025). Mutations in *P53* lead to the loss of its normal function, resulting in abnormal cell proliferation and tumor progression (Gheghiani et al., 2021). Additionally, *P53* mutations often lead to the formation of protein aggregates, resulting in a gain-of-function effect that impairs its normal activity (Blagosklonny, 2000). Mutant p53 exerts a dominant-negative effect by preventing wild-type p53 from binding to its target gene promoters (Willis et al., 2004). The status of p53 is closely linked to the sensitivity of cancer cells to therapeutic drugs (Kim et al., 2013). Therefore, understanding the impact of *P53* mutations on the efficacy of anticancer drugs is crucial for optimizing treatment strategies.

High expression of PLK1 plays a critical role in driving tumorigenesis (Gheghiani et al., 2021) and is closely associated with tumor initiation, progression, and poor prognosis (Liu et al., 2017). PLK1 inhibitors exert their effects by suppressing the function of PLK1, thereby inhibiting tumor cell proliferation and inducing apoptosis (Wissing et al., 2013). Several PLK1 inhibitors have been extensively studied, and BI6727, in particular, has entered phase III clinical trials, offering new hope for cancer treatment strategies (Gutteridge et al., 2016). Despite promising clinical results of BI6727 in other solid tumors, its clinical efficacy in prostate cancer has been underwhelming (Schöffski et al., 2012). Therefore, it is important to understand the factors that influence the efficacy of PLK1 inhibitors in prostate cancer. PLK1 and p53 are closely related and can mutually influence and regulate each other in different ways (Meitinger et al., 2024; Ando et al., 2004). When investigating the factors that influence the efficacy of PLK1 inhibitors, some studies suggest that cells with p53 deficiency are more susceptible to PLK1 inhibitors, leading to apoptosis (Guan et al., 2005; Degenhardt et al., 2010; Sur et al., 2009); however, other studies suggest that the presence of p53 may increase the sensitivity of tumor cells to PLK1 inhibitors (Sanhaji et al., 2012; Sanhaji et al., 2013; Van den Bossche et al., 2019). At present, the role of p53 in modulating the effects of PLK1 inhibitors in tumor cells remains inconsistent.

In this study, we investigated the differential effects of PLK1 inhibitors on prostate cancer cells with different p53 status. We selected three prostate cancer cell lines with different p53 status: PC3 cells, which lack of p53 expression; LNCap cells, which express low levels of wild-type p53; and DU145 cells, which overexpress mutant p53 (Figure 1A) (Isaacs et al., 1991). The PLK1 inhibitor BI6727 inhibits proliferation and induces apoptosis in all three prostate cancer cell lines, consistent with previous studies (Shin et al., 2019). However, the IC50 of BI6727 is lower in LNCaP cells than in PC3 cells (Figures 1B, 2A), which we hypothesize may be related to the presence of functional wild-type p53 in LNCaP cells. To test this hypothesis, PC3 cells lacking of p53 expression were transiently transfected with wild-type p53 or used to construct a stable p53-expressing PC3 cell line for further validation. It was found that p53 enhanced the sensitivity of PC3 to BI6727(Figures 1D–G). In contrast, in LNCaP cells expressing wild-type p53, knockdown of p53 significantly reduced BI6727-induced apoptosis (Figure 2E). Both experiments in PC3 and LNCaP indicate that wild-type p53 affects the sensitivity of prostate cancer cells to BI6727 and promotes apoptosis. It is reported *P53* is mutated in prostate cancer specimens. It is important to investigate whether the mutant forms of *P53* affect the treatment of BI6727. DU145 cells which harbor p53 mutations and express two different p53 mutants, p53P223L and p53V274F(31), were the good models to illustrate this question. Both of these mutations are located in the DNA binding domain. V274F affects DNA binding (type I), while P223L alters structural stability (type II), together impairing both structure and function. These mutations reduce p53 transactivation activity and fail to activate targets like p21 and BAX (Butera and Amelio, 2025; Morton et al., 2017). When p53 was knocked down in DU145 cells, the BI6727-induced apoptosiswas significantly enhanced (Figures 3C,D). Mutant p53 is overexpressed and accumulated in cancer cells, which not only eliminates the activity of wild-type p53 but also tends to acquire oncogenic functions and interfere with p53-independent apoptosis (Sigal and Rotter, 2000). Thus, the p53 mutation in DU145 cells exerts a dominant-negative effect (Kogan-Sakin et al., 2011), with the accumulation of mutant p53 inhibiting BI6727-induced apoptosis in tumor cells. This provides a clear explanation for the discrepancies observed between DU145,PC3 and LNCap cells. By analyzing the p53 mutation status in clinical prostate cancer specimens, we found that p53 mutations are prevalent in prostate cancer (Figure 4). Common p53 missense mutations include R175, Y220, G245, R248, R249, R273, and R282. Among them, R273H and R248Q are DNA-contact mutations, similar to DU145's V274F. R175H, Y220C, R282W, G245S, and R249S are structural mutations, akin to DU145's P223L. Notably, structural mutations are more prevalent (Kennedy and Lowe, 2022; Chen et al., 2022). Overall, the p53 mutations in DU145 represent the most common types of p53 mutations.BI6727 shows better therapeutic efficacy in p53 wild-type cancer cells compared to p53-mutant cells. Therefore, determining the p53 mutation status prior to the application of the PLK1 inhibitor BI6727 may provide a more accurate prediction of the drug’s efficacy. This study utilized cell lines with different p53 statuses, including those with long-term p53 loss, filling gaps in previous research. We further demonstrated that the correlation between p53 and BI6727 activity is not a general stress response. BI6727 specifically inhibits the ubiquitination of wild-type p53 by preventing Topors degradation, leading to p53 accumulation (Figure 5). Unlike other PLK1 inhibitors, BI6727 has minimal impact on global protein ubiquitination (Wang et al., 2024).However, a limitation of this study is that we did not investigate the specific mechanisms by which p53 mutations affect the efficacy of BI6727 in DU145 cells.

p53 has different mutation rates in different stages of prostate cancer. Cellularly, LNCaP, a hormone-dependent prostate cancer cell, expresses wild-type p53; whereas, PC3 and DU145, hormone-independent prostate cancer cells, loses p53 expression and expresses mutant-type p53, respectively. Clinically, it has been reported that the mutation rate of p53 is 20% in primary castration-naive prostate cancer; 37% in metastatic castration-naive prostate cancer; and 73% in metastatic castration -resistant prostate cancer. Our results show that the wild type p53 promotes the sensitivity of prostate cancer cells to BI6727. Therefore, it might be that BI6727 would be even less effective in treating late-stage prostate cancer (Hamid et al., 2019; Teroerde et al., 2021).

In summary, this study used three types of prostate cancer cell lines with different status of p53 to investigate the p53-PLK1 inhibitor BI6727. Wild-type p53 makes prostate cancer cells more sensitive to the effects of BI6727, while mutant p53 exerts a dominant-negative effect and suppresses the sensitivity of prostate cancer cells to BI6727. As p53 mutations are common in prostate cancer patients, it might be one of important reasons why BI6727 shows minimal anti-tumor activity in phase I trials in prostate cancer.

## Data Availability

The original contributions presented in the study are included in the article/supplementary material, further inquiries can be directed to the corresponding authors.

## Abbreviations

IC50, half‐maximal inhibitory concentration; PARP, poly (ADP)ribose polymerase; PLK1, Polo‐like kinase 1; siRNA, small‐molecule interfering RNA.
